# Enorme kyste amygdaloïde cervical: à propos d'un cas

**DOI:** 10.11604/pamj.2018.31.147.17028

**Published:** 2018-10-26

**Authors:** Ahmed Rouihi, Bouchaib Hemmaoui, Noureddine Errami, Fouad Benariba

**Affiliations:** 1 Service d'ORL et de CCF de l'Hôpital Militaire d'Instruction Mohamed V de Rabat, Faculté de Médecine et Pharmacie Rabat, Université Mohamed V Rabat, Rabat, Maroc

**Keywords:** Appareil branchial, kyste amygdaloïde, chirurgie, Branchial apparatus, amygdaloidal cyst, surgery

## Abstract

Les kystes amygdaloïdes sont des tumeurs bénignes kystiques dysembryologiques qui se développent au niveau de la partie antéro-latérale du cou, ils représentent 2% des tumeurs latéro-cervicales du cou, ils comptent parmi les plus fréquentes des anomalies branchiales, ils représentent 6,1 à 85,2% des anomalies de la deuxième fente. Ils sont dus à la persistance du sinus cervical au cours de la différenciation de l'appareil branchial. Ils se manifestent par une tuméfaction latéro-cervicale située au bord antérieur du muscle sterno-cléido-mastoïdien. Leur nature kystique est confirmée par l'échographie et la TDM. Le traitement consiste à l'exérèse chirurgicale. Nous rapportons le cas d'une femme de 24 ans qui a consulté pour une énorme tuméfaction latéro-cervicale gauche qui évolue depuis 3 ans sans autres symptomatologies associées. Une cervicotomie exploratrice avec étude anatomo-pathologique ont été réalisées, le diagnostic histologique retenu était un kyste amygdaloïde sans signes de malignité. L'objectif de ce travail est d'analyser les caractéristiques anatomo-cliniques et discuter des modalités de prise en charge et les indications thérapeutiques de cette affection.

## Abstract (en)

Amygdaloid cysts are benign dysembryologic cystic tumors developing in the anterolateral portion of the neck. They account for 2% of all neck tumors located in the laterocervical region. They mostly result from branchial cleft abnormalities, accounting for 6.1-85.2% of all second branchial cleft abnormalities. They are due to the persistence of the cervical sinus during the differentiation of the branchial apparatus. They manifest as a laterocervical swelling in the anterior edge of the sternocleidomastoid muscle. The diagnosis of cyst is confirmed by ultrasound and CT scan. Treatment is based on surgical resection. We here report the case of a 24-year old woman presenting with voluminous left laterocervical swelling evolving over the past 3 years without any associated symptoms. Exploratory cervicotomy with anatomopathological examination were performed. The histologic diagnosis of amygdaloid cyst with no sign of malignancy was retained. This study aims to analyze the anatomoclinical features and to discuss the therapeutic options for the management of this disease.

## Introduction

Les kystes amygdaloïdes comptent parmi les plus fréquentes des anomalies branchiales, ils représentent 6,1 à 85,2% des anomalies de la deuxième fente. Ils sont dus à la persistance du sinus cervical au cours de la différenciation de l'appareil branchial. Le siège habituel est le tiers moyen du bord antérieur du muscle sterno-cléido-mastoïdien mais ils peuvent se situer à n'importe quel point depuis le muscle constricteur moyen du pharynx à la région susclaviculaire. Nous rapportons cette observation rare d'un énorme kyste amygdaloïde latéro-cervical.

## Patient et observation

**Figure 1:**
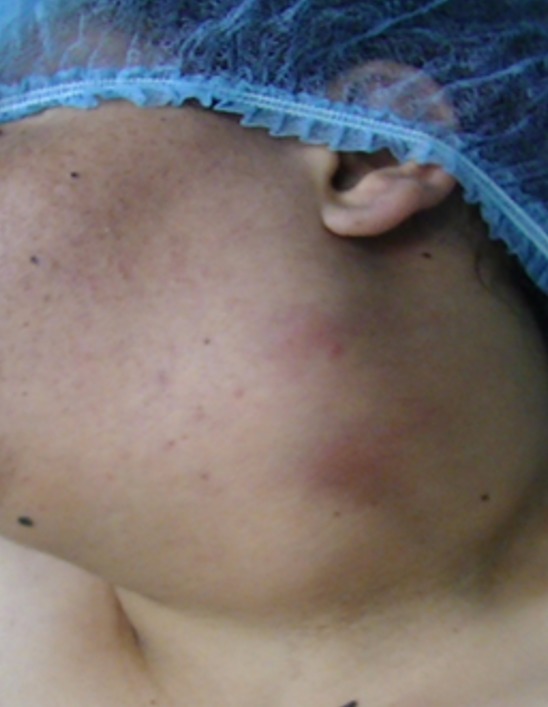
photo de la patiente montrant le kyste amygdaloïde cervical

**Figure 2:**
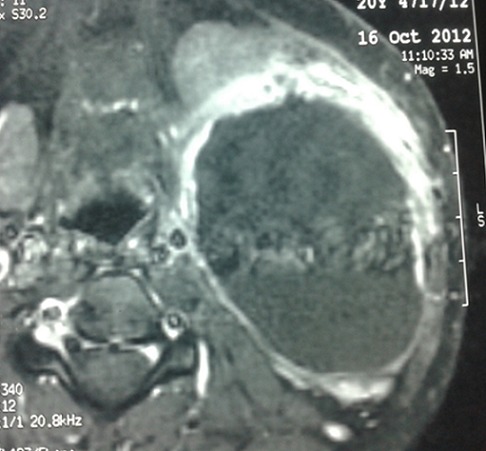
TDM cervico-faciale en coupe axiale: formation kystique bien limitée avec prise de contraste en périphérie

**Figure 3:**
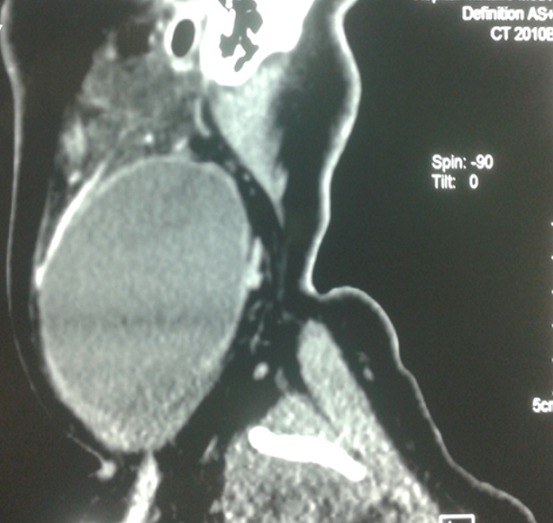
IRM cervicale coupe sagittale montrant une formation kystique en hyposignal T2

**Figure 4:**
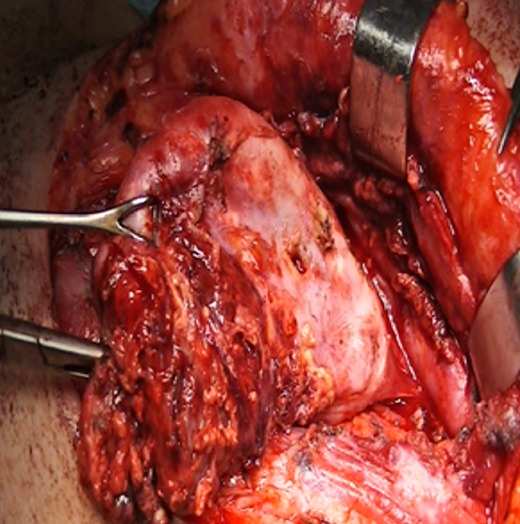
photo montrant l'exérèse chirurgicale du kyste

**Figure 5:**
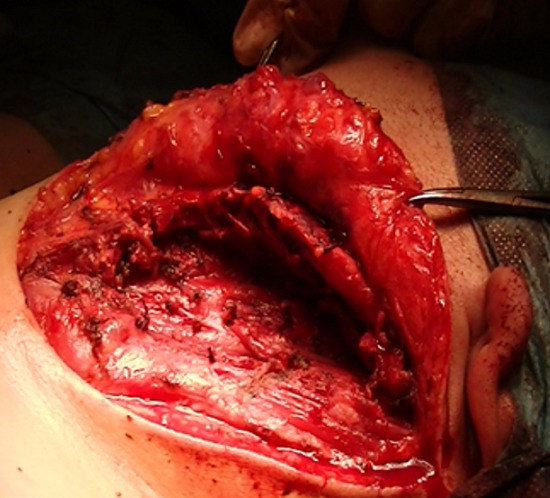
photo montrant le site opératoire après l'exérèse chirurgicale du kyste

Une patiente de 24 ans, sans antécédents pathologiques notables, qui a présenté depuis 3 ans une tuméfaction latéro-cervicale gauche qui augmente de volume progressivement, sans signes otologiques ou rhinologiques associés, l'examen clinique a retrouvé une énorme tuméfaction latéro-cervicale gauche allant de la pointe de la mastoïde en haut jusqu'à la clavicule en bas, indolore, peu mobile, non pulsatile mesurant presque 12cm/4cm, de consistance rénitente, sa limite parfonde était impossible à préciser, la peau en regard était légèrement inflammatoire. L'examen de l'oropharynx, du rhinopharynx et du pharyngolarynx était normal (Figure 1). La TDM cervico-faciale injectée a objectivé la présence d'une volumineuse formation latéro-cervicale gauche isolée hétérogène étendue de l'angle mandibulaire jusqu'à la région sus claviculaire qui refoule l'axe jugulocarotidien gauche en dedans et le muscle sternocléidomastoïdien en dehors et qui mesure 10x7x7cm sans adénopathies satellites (Figure 2). L'IRM cervico-faciale a retrouvé une volumineuse formation latéro-cervicale gauche isolée étendue de l'angle mandibulaire jusqu'à la région sus claviculaire qui refoule l'axe jugulo-carotidien gauche et le muscle sternocléidomastoïdien de même dimensions (Figure 3). Le diagnostic d'un kyste cervical a été retenu, La patiente a bénéficié d'une cervicotomie gauche avec une résection complète du kyste, les suites opératoires étaient simples. L'examen anatomopathologique a confirmé le diagnostic d'un kyste amygdaloïde sans signes de malignité (Figure 4, Figure 5).

## Discussion

Les kystes et les fistules congénitaux de la face et du cou sont des malformations d'origine embryologique peu fréquentes et mal connues. L'ORL doit reconnaître précocement ces lésions pour permettre une prise en charge adaptée [1-3]. Les kystes amygdaloïdes ou lympho-épithélials ou kystes du sinus cervical sont des tumeurs bénignes kystiques dysembryologiques rares qui correspondent à des défauts de résorption du deuxième arc branchial [2, 4] et qui se développent au niveau de la partie antéro-latérale du cou. La fréquence des kystes amygdaloïdes par rapport aux anomalies de la deuxième fente varie de 6,1 à 85,2% [1, 2]. L'âge de découverte est plus avancé que pour les autres anomalies congénitales, avec deux pics de fréquence, l'enfant de moins de 5 ans et entre la 2^ème^ et la 3^ème^ décade, sans aucune prédominance du sexe.

Sur le plan clinique, un kyste du sinus cervical apparaît sous la forme d'une tuméfaction ovalaire, rénitente, mobile sous les plans superficiels, le plus souvent située à proximité de la bifurcation carotidienne en position sous-hyoïdienne [4, 5]. Bien que ces lésions soient congénitales, elles ne sont généralement identifiées qu'entre la seconde et la quatrième décennie de la vie, lorsqu'elles augmentent de taille ou deviennent symptomatiques; il communique parfois avec la peau ou le pharynx [2] spontanément ou suite à une surinfection. La communication avec l'extérieur se fait par un canal étroit que l'on appelle fistule cervicale externe, dont l'orifice externe se situe souvent à la réunion du tiers moyen et tiers inférieur du bord antérieur du muscle sterno-cléido-mastoïdien.

La TDM ou l'IRM sont particulièrement indiquées pour différencier la lésion des autres tumeurs parapharyngées: un hémangiome, lymphangiome ou kyste dermoïde, adénopathie métastatique dont la distinction avec un kyste amygdaloïde dégénéré ou une métastase intra kystique est très difficile et la confirmation reste anatomopathologique après une exérèse chirurgicale [1, 2]. L'imagerie actuelle et particulièrement la résonance magnétique (IRM) confirme la nature kystique et la proximité des gros vaisseaux du cou, sans préjuger du caractère primitif ou secondaire du kyste amygdaloïde malin. La constatation d'une fistule de la deuxième fente, surtout si elle est bilatérale doit faire rechercher un syndrome branchiootorénal par une échographie rénale. Ces kystes ont été classés en quatre stades par Bailey [4]; Type I: kyste superficiel, sous l'aponévrose cervicale superficielle, Type II: kyste sous l'aponévrose cervicale moyenne, en région pré-vasculaire (le plus fréquent), Type III: kyste inter-vasculaire, dans la fourche entre ACI et ACE, Type IV: kyste intra-vasculaire, entre paroi pharyngée et axe carotidien.

Sur le plan histologique, Le kyste amygdaloïde est tapissé par un épithélium de différents types, le plus souvent malpighien [2]; il peut s'agir aussi d'un épithélium de type cylindrique cilié d'origine ectodermique. Certains auteurs pensent que la présence de kératine, la présence de tissu lymphoïde sont des critères obligatoires au diagnostic du kyste amygdaloïde [2]. Le diagnostic différentiel se pose surtout lorsqu'il existe une masse latéro-cervicale isolée sans fistule qui doit faire évoquer chez l'enfant un lymphangiome kystique uniloculaire, un lipome ou une adénopathie [5]. L'infection est la complication la plus souvent révélatrice de cette malformation compliquant son exérèse chirurgicale du kyste. Le kyste amygdaloïde, parfois rapidement évolutif et compressif, peut entrainer des sensations de malaises et des bradycardies par compression rapide et importante du bulbe carotidien, dans ce cas le kyste doit être ponctionné pour soulager le patient. La transformation maligne au sein du kyste branchial est décrite dans la littérature mais elle reste exceptionnelle, 15 cas seulement ont été publiés dans la littérature mondiale dont 4 carcinomes in situ et 11 carcinomes épidermoïdes infiltrant [3]. Le diagnostic de kyste branchial malin doit faire l'objet de plus grandes réserves et ne doit être retenu qu'après avoir éliminé une métastase au sein du kyste branchial d'un carcinome primitif à distance ainsi qu'une simple évolution kystique d'un ganglion métastasique.

## Conclusion

La prise en charge thérapeutique est toujours chirurgicale, elle doit être réalisée le plus tôt possible pour limiter le risque de remaniements inflammatoires liés aux épisodes infectieux, il conviendra alors de n'opérer qu'après refroidissement complet des infections par une antibiothérapie adaptée. On peut repérer le trajet fistuleux en le cathétérisant avec injection de bleu de méthylène. La dissection se poursuit au contact du trajet fistuleux, elle s'arrête rapidement en cas de fistule borgne externe. Si la fistule se poursuit vers le haut, une deuxième incision est nécessaire pour suivre son trajet. La rupture de la fistule à sa partie haute est habituellement sans conséquence, la fistule résiduelle se drainant dans l'oropharynx. Enfin, il n'est pas utile d'effectuer une amygdalectomie systématique [2].

## Conflits d’intérêts

Les auteurs ne déclarent aucun conflit d’intérêts.
